# Comparison of transcriptomic landscapes of bovine embryos using RNA-Seq

**DOI:** 10.1186/1471-2164-11-711

**Published:** 2010-12-17

**Authors:** Wen Huang, Hasan Khatib

**Affiliations:** 1 Department of Dairy Science, University of Wisconsin, Madison, WI 53706, USA

## Abstract

**Background:**

Advances in sequencing technologies have opened a new era of high throughput investigations. Although RNA-seq has been demonstrated in many organisms, no study has provided a comprehensive investigation of the bovine transcriptome using RNA-seq.

**Results:**

In this study, we provide a deep survey of the bovine embryonic transcriptomes, the first application of RNA-seq in cattle. Embryos cultured *in vitro *were used as models to study early embryonic development in cattle. RNA amplified from limited amounts of starting total RNA were sequenced and mapped to the reference genome to obtain digital gene expression at single base resolution. In particular, gene expression estimates from more than 1.6 million unannotated bases in 1785 novel transcribed units were obtained. We compared the transcriptomes of embryos showing distinct developmental statuses and found genes that showed differential overall expression as well as alternative splicing.

**Conclusion:**

Our study demonstrates the power of RNA-seq and provides further understanding of bovine preimplantation embryonic development at a fine scale.

## Background

The recent developments in high throughput sequencing technologies allow for surveys of transcriptomes at unprecedented completeness and resolution. The 'next generation' RNA sequencing technology (RNA-seq) has several advantages, such as the ability to detect unannotated transcriptional activity, to differentiate between different transcriptional or splicing isoforms, and to provide digital measurements at single base resolution. Recently, the efficacy of RNA-seq has been demonstrated in humans [1] and model organisms including yeast [2], *Arabidopsis *[3,4], and mice [5]. An RNA-seq experiment typically sequences millions of cDNA fragments, which are subsequently aligned to a reference genome or assembled *de novo *to recover structural models of annotated and unannotated genes and/or infer expression. Due to its attractive features and continually decreasing cost, RNA-seq is quickly replacing traditional technologies such as microarrays for high throughput transcriptomic studies.

The recent sequencing and analysis of the bovine genome have provided opportunities for further post-genomic investigations of this important livestock species [6]. Despite the increasingly wide applications of RNA-seq, its use in cattle had not yet been reported. In this study, we present the first application of RNA-seq in cattle by sequencing RNA from embryos cultured *in vitro*. Because early embryonic loss accounts for the majority of cow infertility [7], we have previously created an *in vitro *fertilization (IVF) system to recapitulate the early development of cattle and allow systematic investigations of abnormities during the very early stages of development [8]. The transcriptomes of IVF embryos showing distinct developmental statuses were previously profiled using microarrays, and few gene expression differences were detected in embryos undergoing abnormal versus normal development [9].

Expression microarrays, however, rely on existing genome annotations and lack the ability to detect more complex regulations in gene expression such as changes in alternative splicing. Therefore, in this study, we seek to take advantage of RNA-seq to identify transcriptomic changes associated with abnormal bovine early embryonic development, especially for transcriptional activities that have not been annotated before and differential alternative splicing that could not be detected by expression microarrays. Although the importance of alternative splicing in regulating complex traits has been long recognized and extensively studied, there is limited information on the pattern of alternative splicing in cattle or its roles in bovine embryonic development.

Herein, using RNA-seq, we estimated and compared digital gene expression in IVF embryos. In addition, unannotated transcribed regions in the bovine genome and novel splice junctions were discovered. Finally, we show that alternative splicing is widespread in the bovine embryonic transcriptome, and we have identified alternative splicing events associated with abnormal bovine embryonic development. This study is the first application of RNA-seq in cattle and provides fine-scale insights into bovine preimplantation embryonic development.

## Results

### Massively sequencing the bovine embryonic transcriptome

To overcome the scarcity of RNA present in individual embryos, we linearly amplified RNA [10] from two pools of embryos: one comprised 20 embryos that properly completed embryonic development to blastocyst stage (hereafter referred to as 'blastocysts'); the other consisted of 20 embryos that showed retarded morphology by the same developmental time point (hereafter referred to as 'degeneratives'). The amplified RNA (aRNA) was complementary to the polyadenylated RNA species and considered equivalent to polyA+ RNA. The aRNA samples were sequenced according to Illumina's mRNA-seq protocol on a Genome Analyzer IIx. Approximately 35 million fragments were sequenced (single or paired end reads) for both blastocysts and degeneratives (Table 1).

**Table 1:** Summary of sequencing read alignment to the reference genome

Sample	Blastocysts	Degeneratives
Paired end reads	18,377,462 × 2	20,499,571 × 2

Single end reads	17,597,407	13,770,885

Total sequenced fragments	35,974,869	34,270,456

Fragments mapped to nuclear genome	22,511,851	21,480,971
(percent mapped)	(62.6%)	(62.7%)

Uniquely mapped fragments	20,417,798	19,830,476
(percent uniquely mapped)	(90.7%)	(92.3%)

Fragments mapped to mitochondrial genome	3,690,752	2,188,146

Fragments mapped to annotated junctions	2,174,923	1,268,315
(number of junctions)	(65,743)	(62,788)

Fragments mapped to putative novel junctions	129,851	84,600
(number of junctions supported by at least 2 fragments)	(10,638)	(8,686)

Fragments mapped to annotated exons	11,596,019	7,103,823

Fragments overlapped with annotated introns	2,525,630	3,644,395

Fragments mapped to annotated genes (exons + introns)	14,121,649	10,748,218

Fraction of intronic fragments among genic fragments	17.9%	33.9%

PolyA containing reads	534,090	862,915

Mapped polyA reads (with polyA signal)	112,170	120,552
(number of putative polyA sites)	(11,389)	(15,493)

Sequencing reads were mapped to the reference genome (Btau4) using the software package Tophat [11]. Approximately 60% of total sequenced fragments could be mapped contiguously or to exon-exon junctions of the nuclear genome, among which over 90% were mapped uniquely (Table 1). Reads that could not be mapped initially by Tophat were examined for evidence of polyA tails, trimmed to contain non-polyA sequences, and mapped to the reference genome using the software package Bowtie [12]. In total, 226,735 and 287,088 polyA containing fragments could be mapped uniquely in blastocysts and degeneratives respectively, corresponding to 11,389 and 15,493 putative polyA signals (AAUAAA or AUUAAA) within 50 bases of the cleavage sites.

Among the uniquely mapped fragments, 14,121,649 and 10,748,218 were mapped to annotated genes (exons and introns) in blastocysts and degeneratives, respectively, including 2,174,923 and 1,268,315 fragments mapped to annotated exon-exon junctions (Table 1). We noticed a marked difference between the fractions of intronic fragments in blastocysts (17.9%) and degeneratives (33.9%; Table 1). This observation will be dicussed in more detail below. Moreover, the substantially smaller number of junction fragments in degenerative embryos was likely a result of a higher fraction of fragments overlapped with introns (Table 1). Nevertheless, using Tophat, a total of 10,638 and 8,686 novel splice junctions supported by at least two fragments were discovered in blastocysts and degeneratives respectively, among which 8,315 were found in both samples (Table 1).

### Digital gene expression estimated by RNA-seq

**Figure 1:**
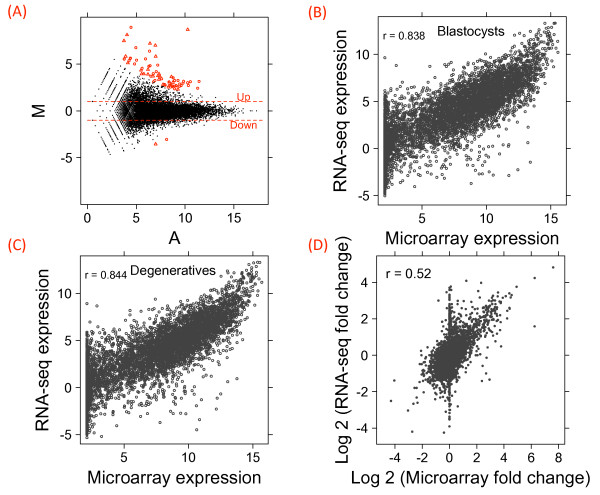
**Digital gene expression using RNA-seq**. Differential gene expression was analyzed using the "DESeq" R package and plotted as a MA plot (a). Each point represents a gene (circle) or novel transcribed unit (triangle). The "M" axis is the log 2 fold change as compared to blastocyst and the "A" axis is the average log 2 normalized counts in the two samples. Red points indicate significant change as called by "DESeq" at FDR = 0.01. The two horizontal dashed lines marked the two-fold change thresholds. For comparison between RNA-seq and microarray, log 2 transformed RNA-seq and microarray gene expressions were plotted for blastocyst (b) and degenerative (c) respectively. Fold changes estimated using RNA-seq and microarrays were also compared (d).

We next obtained digital gene expression values by counting aligned fragments entirely overlapped with constitutive exons. To enable comparison with microarray data obtained in a previous study [9], only overall expressions of 19,043 annotated protein coding genes were obtained to this end. Among all genes analyzed, 16,327 and 16,380 were overlapped by at least one sequencing fragment in blastocysts and degeneratives, respectively. Raw sequencing fragment counts per gene were quantile normalized, which has been shown to reduce bias when comparing gene expression between samples [13]. Differentially-expressed genes were called at a false discovery rate (FDR) of 1% using the DESeq package in R [14], which models count data by a negative binomial distribution. Similar to our previous findings [9], overall gene expression in degenerative embryos was largely the same as in blastocysts. The correlation between blastocysts and degeneratives was as high as 0.986 and relatively few genes (n = 47) were differentially expressed (Figure 1a, Additional File 1). Because no replication was performed, the statistical significance could only represent differences in these two particular samples. Nevertheless, because 20 embryos were pooled to generate each of the sequencing libraries, the gene expression measurements represented averages across 20 embryos.

To evaluate the RNA-seq gene expression measurements, we compared them to normalized expression microarray measurements for 6,169 genes that could be uniquely identified on both platforms. RNA-seq was particularly advantageous for low-abundance transcripts, for which microarrays are generally insensitive (Additional File 2). The correlations between the two platforms were high for both blastocysts (r = 0.838, Figure 1b) and degeneratives (r = 0.844, Figure 1c). Moreover, the correlation between fold changes on two platforms was also high (r = 0.525, Figure 1d). Interestingly, we observed a clear curvature towards the RNA-seq axis when comparing RNA-seq and microarray gene expressions (Figures 1b and 1c), suggesting that microarrays likely underestimated expressions of genes that were most highly expressed.

### Gene Ontology enrichment analysis

To gain insights into the biological processes that are regulated during abnormal embryonic development, we tested for enrichment of differentially expressed genes in gene ontology (GO) terms. Genes that showed a nominal significance of p = 0.05 and had GO annotations (n = 330) were selected and tested against the background set of all genes with GO annotations (n = 8139). We found several GO terms significantly enriched (FDR = 0.01) for differentially expressed genes, among which were GO processes related to developmental processes (Table 2).

**Table 2:** GO terms enriched for differentially expressed genes

GO term	Expected count	Observed count	P value	FDR
Biological Process				
Multicellular organismal process	40.9	78	1.73E-09	4.77E-06
System development	25.7	53	1.44E-07	1.99E-04
Negative regulation of biological process	17.7	38	4.13E-06	3.79E-03
Cellular developmental process	21.0	42	7.63E-06	4.21E-03
Tissue development	8.7	23	1.52E-05	6.98E-03
Cellular Component				
Proteinaceous extracellular matrix	4.9	17	5.57E-06	3.84E-03

### Identification and analysis of novel transcribed units

One important feature of RNA-seq is its independence from gene annotations, which allows investigating unannotated transcriptional activities. Uniquely mapped alignments were assembled using the software package Scripture [15], and the assembled transcripts were compared to known gene models to look for transcriptional activities in intergenic regions. We have defined novel transcribed units (TUs) as regions containing transcripts that were 1) at least 1000 bp away from known gene boundaries; 2) of length ≥ 500 bp; 3) covered by at least 40 fragments per kb (about 5 fragments per base); and 4) <50% repetitive sequence. We found 1,272 and 1,348 novel TUs meeting these criteria in blastocysts and degeneratives, respectively (Table 3). Importantly, a substantial fraction of novel TUs was supported by confident polyA-containing fragments, suggesting that the transcripts were potentially polyadenylated (Table 3).

**Table 3:** Identification of novel transcribed units (TUs) by RNA-seq

Sample	Total	Supported by polyA reads	Supported by EST alignment (>50%)
Blastocyst	1,272	532	877
Degenerative	1,348	531	804

**Figure 2:**
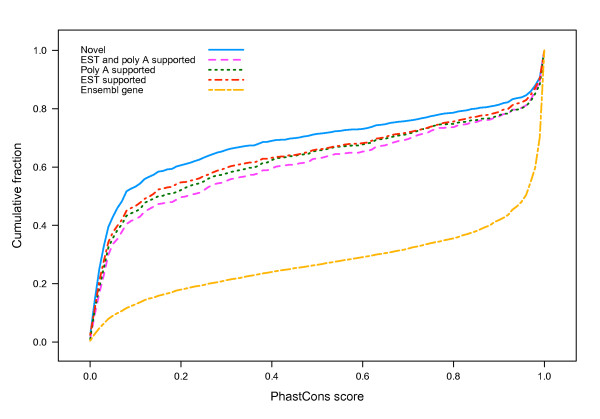
**Cumulative distribution of phastCons scores for known genes and novel TUs supported by various sources of evidence**.

We then looked for evidence to support the novel TUs found by our RNA-seq experiment from external sources. EST alignments were downloaded from UCSC genome browser (http://genome.ucsc.edu/) and compared to novel TUs. About two thirds of the novel TUs in both blastocysts and degeneratives were supported by EST alignments. In addition, phastCons scores [16] calculated from the 5-way alignment of five mammalian species (cow, dog, human, mouse and platypus) were also obtained for all novel TUs and annotated genes. Although novel TUs appeared to be less conserved than annotated genes distributionally, more than 20% of them exhibited very conserved mean phastCons scores, especially those supported by polyA reads and EST alignments (Figure 2).

**Figure 3:**
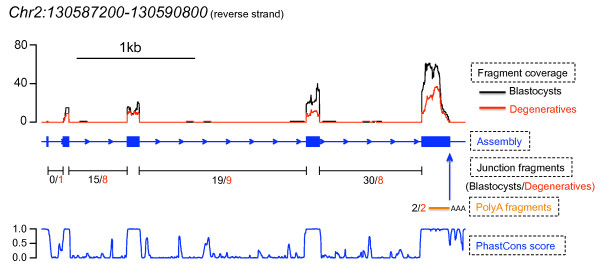
**An example of identified novel TUs**. Fragment coverage, the reconstructed transcript model by Scripture, numbers of junction fragments, numbers of polyA fragments, and phastCons scores were plotted from top to bottom. Blastocysts are in black and degeneratives are in red.

A TU located on chromosome 2 that met our definitions of a novel TU is shown as an example in Figure 3. This novel TU was supported by polyA fragments and EST alignments and showed remarkable conservation among mammals. Furthermore, a blast search of the mRNA sequence against the human RefSeq database also revealed a highly similar transcript coding for the ZDHHC18 protein.

In total, we have identified 1,648,307 transcribed bases in a combined total of 1,785 regions outside gene boundaries that have not been annotated, indicating that the current bovine genome annotation is incomplete. To test for differential expression between blastocysts and degeneratives for these novel TUs, raw fragment counts from novel TUs and annotated genes were combined, quantile normalized, and analyzed using the "DESeq" package. We found 25 novel TUs significantly altered in degeneratives (Figure 1a, Additional File 1). One particularly interesting example of differentially expressed novel TUs was located on the X chromosome, which showed almost exclusive expression at a high level in degeneratives (normalized counts of 60 and 24,892 in blastocysts and degeneratives, respectively).

### Analysis of alternative splicing

We utilized the RNA-seq data to characterize and compare the patterns of alternative splicing in blastocysts and degeneratives. We first asked how frequently alternative splicing occurred in IVF embryos. Because detection of alternative splicing events is critically limited by the number of junction fragments, we first combined the datasets to increase the sensitivity of detection. We detected a total of 4,426 "exon skipping", "alternative 5' splice site", and "alternative 3' splice site" in 2,032 multi-exon genes in the combined dataset (Table 4), suggesting that alternative splicing is widespread in preimplantation bovine embryos.

**Table 4:** Alternative splicing (AS) events and genes detected by RNA-seq

AS type	Number of AS events detected			Number of genes undergoing AS		
	
	Combined dataset	Blastocysts	Degeneratives	Combined dataset	Blastocysts	Degeneratives
Exon skipping	2,892	1,747	1,252	1,625	1,067	887
Alternative 5' splice site	662	419	259	421	268	186
Alternative 3' splice site	872	539	372	772	438	330

Total	4,426	2,705	1,883	2,032	1,566	1,277

**Figure 4:**
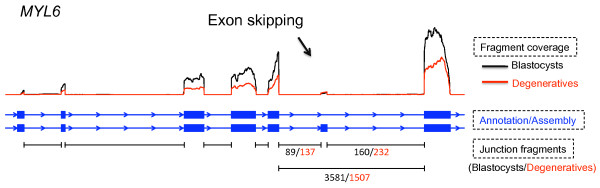
**Example of significant differential alternative splicing events**. Fragment coverage, annotated and reconstructed gene models (in this case, they are the same), and numbers of junction fragments were plotted from top to bottom.

The numbers of detected intra-sample alternative splicing events were substantially smaller (Table 4), primarily due to fewer junction fragments and sample specific alternative splicing. The absolute numbers of detected alternative splicing events in separate samples should not be compared directly as they were confounded by the numbers of junction fragments in these datasets. Instead, we tested differential alternative splicing using Fisher's exact test for all events that can be detected in the combined dataset. Among the 2,892 "exon skipping" events tested, 23 were significantly associated (p < 0.01) with embryonic developmental status; among those, two were still significant after multiple testing correction and controlling FDR at 1%. For example, the sixth exon of the *MYL6 *(myosin light chain 6) gene showed a significantly higher inclusion ratio (OR = 3.51, p = 3.65 × 10^-26^) in degeneratives than in blastocysts (Figure 4). Similarly, three "alternative 5' splice site" and 11 "alternative 3' splice site" events showed nominal significance (p < 0.01), yet none of them retained significance after multiple testing correction.

## Discussion

In this study, we present the first application of RNA-seq in cattle using IVF embryos. We show the high potential of RNA-seq in transcriptomic studies, particularly in organisms that are not fully annotated. We found unannotated genes that may play roles in early embryonic development and several genes that showed differential alternative splicing between blastocysts and degeneratives. Our findings clearly demonstrate the power of RNA-seq and provided further insights into bovine early embryonic development at a finer resolution.

RNA-seq has been frequently cited as more advantageous than microarrays in many aspects [17]. Indeed, with a typical sequencing depth, we were able to obtain digital expression measures for many low-abundance genes, for which expression microarrays do not have sufficient sensitivity (Additional File 2). Perhaps a more important feature of RNA-seq, particularly for incompletely annotated genomes such as cattle, is its independence from existing annotations. Applying stringent criteria, we were able to identify a combined total of 1,785 novel TUs covering more than 1.6 million bases in intergenic regions that have not been annotated before. Interestingly, a substantial fraction of these novel TUs were differentially expressed (Additional File 1), including a TU that showed almost exclusive expression in degenerative embryos. This X chromosome linked transcript (chrX:8564208-8564984) was supported by EST alignments showing the same gene structure. It is located in a region that lacks conservation among mammals and was predicted to have the potential to code for a 134 amino acid protein. RNA-seq is believed to have a higher dynamic range than microarrays, partly because microarrays often have detection limits. When comparing gene expression measures on the same sample for both platforms, we observed a clear curvature towards the RNA-seq axis as expression increased (Figure 1), indicating that microarray measurements were likely biased for highly-expressed genes, leading to a lower dynamic range. However, the agreement between fold changes on the two platforms was high, and we did not detect a systematic decrease in fold change estimates by microarrays (Figure 1).

Another important advantage of RNA-Seq is its ability to detect alternative splicing events. Indeed, we detected thousands of alternative splicing events and tested for their association with the status of embryonic development (Table 4). Among the differentially spliced genes between blastocysts and degenratives, *MYL6 *showed a higher inclusion ratio of its sixth exon in degeneratives, which was validated experimentally using real time RT-PCR. The inclusion and exclusion of exon six of the *MYL6 *gene switch between the smooth muscle isoform and the non-muscle isoform of myosin light chain, respectively [18]. In degeneratives, the smooth muscle isoform expressed at a higher ratio than in blastocysts. Importantly, the protein and nucleotide sequences, as well as the alternative splicing pattern, are conserved in mammals. Furthermore, gene expression of *MYL6 *has been previously shown to be associated with bovine blastocyst formation, suggesting an important role of this gene in preimplantation embryonic development in cattle [19].

The differentially expressed novel TUs and differential alternative splicing events identified in this study adds additional layers of information to transcriptomic changes associated with abnormal embryonic development, especially for the bovine genome that has incomplete annotation and limited information on alternative splicing.

Meanwhile, we also asked if we were able to identify differentially expressed genes or pathways that were previously known to affect early embryonic development. For example, *IGFBP7 *(ENSBTAG00000019368) was upregulated by about eight fold in degenerative embryos significantly (Additional File 1). Importantly, this protein has been shown to promote apoptosis [20] and may have played a role in the embryonic degeneration process. Moreover, we found an enrichment of differentially expressed genes located to extracellular matrix (Table 2). This result, together with previous findings on the possible involvement cell communication and cell adhesion in early embryonic development [9], suggested that defective communication between cells may play an important role in the abnormal development of degeneratives. Indeed, many of the detected differentially expressed or spliced genes do not have known roles in embryonic development. The identification of such novel candidate genes would necessitate further investigation.

The linear amplification of mRNA was necessary to obtain sufficient materials to generate sequencing libraries from embryos. We were able to amplify approximately 2 ug aRNA from 100 ng total RNA. Assuming a 2% mRNA fraction, this was about 1000-fold amplification, providing a convenient approach for RNA-seq with limited starting materials. However, the effect of mRNA amplification on digital gene expression measurements by RNA-seq is not known. As expected, we observed higher coverage towards the 3' ends of transcripts in RNA-seq (e.g., Figures 3 and 4) as well as in probe hybridization in the microarray data. This also limited our ability to reconstruct full length transcripts, particularly for those expressed at low level. More systematic investigation is required to assess how this artificial shortening of transcripts during mRNA amplification may affect RNA-seq results.

We observed a substantial fraction of intronic reads in both embryo samples. Meanwhile, degeneratives contained considerably more intronic reads (Table 1). In addition, we did not detect any particular subset of genes that preferentially contained intronic reads in degenerative embryos. The evidence on the distribution of sequencing reads in the genome in RNA-seq was not consistent from other studies. Using polyA-selected RNA from liver total RNA, Mortazivi et al. [5] reported 93% and 3% of mapped reads aligned to exons and introns, respectively. In contrast, Marioni et al. [17] observed 32% genic reads mapped to introns, using liver and kidney RNA samples and similar sequencing protocols as Mortazavi et al.[5]. There are several possible explanations for the high fraction of intronic reads in our degenerative embryo sample. First, minimal DNA contamination may be fragmented, selected, and PCR enriched during library generation, though the chance was likely small, particularly because RNA had been amplified (or selected for a typical RNA-seq) and the fragmentation protocol in RNA-seq specifically targeted RNA. To test for possible DNA contamination in our aRNA samples experimentally, we randomly selected several intergenic and intronic genomic regions, and the results indicated no DNA contamination at detectable level by PCR (Additional File 3). Second, nuclear pre-mRNA containing polyA tail may also be purified, amplified, and sequenced. It is important to point out that introns contain many more bases than exons thus a slight increase in pre-mRNA fraction may increase intronic reads disproportionally. Finally, alternative splicing events that retain introns may also increase the fraction of intronic reads. However, this would require many genes or highly-expressed genes to undergo such alternative splicing, which was not observed in our data.

Because a slight increase in the pre-mRNA fraction may result in dramatic increase in intronic reads (Additional File 4), for RNA-seq experiments that contain a substantial fraction of intronic reads, normalization by total mapped reads may severely bias gene expression estimates. For example, normalization by total mapped reads such as reads per kilobase per million mapped reads (RPKM) may underestimate gene expression if the intronic fraction is higher because there would be fewer reads on exons but the total reads remain the same. In addition, total read counts can also be dramatically affected by a few genes that express at a very high level. For these reasons, we chose to quantile normalize raw read counts, which is less affected by total read counts and has been shown to reduce bias for differential gene expression analysis [13].

Our results indicated that the current bovine genome annotation is far from complete. In addition to a combined total of 13,938 novel junctions, we also discovered 1,785 novel TUs. Although RNA-seq is able to detect unannotated transcriptional activities, various filters are needed to avoid detection of spurious "genes". Several software packages have been developed to identify novel splice junctions [11] and reconstruct transcripts from RNA-seq data [15,21], yet sufficient sequencing depth and length are required. Manual inspection of reconstructed transcripts in our data showed that the accuracy was significantly affected by false splice junctions and low coverage. As sequencing technologies evolve to longer and more accurate reads, and more powerful software packages are developed, the need for a complete annotation is further limited. Nonetheless, a more complete annotation of the bovine genome will likely benefit RNA-seq at the current point.

Despite RNA-seq providing a deep survey of the transcriptomes of IVF embryos, little difference in overall gene expression was detected in degenerative embryos. Nevertheless, preference for a particular spliced isoform was observed for several genes in degeneratives. In addition, the frequent occurrence of alternative splicing in IVF embryos provided a repository for regulation of gene expression. Although we sequenced at standard depth (e.g., one lane per sample), it became apparent that deeper sequencing was required to detect subtler changes in alternative splicing with sufficient statistical power.

## Conclusions

In summary, we demonstrated RNA-seq in cattle, providing for the first time a high resolution map of transcriptional and alternative splicing activities in IVF embryos. The results provided further systematic understanding of mammalian embryonic development at a fine scale.

## Methods

### Sample collection and sequencing

IVF embryos were collected as previously described [9]. Briefly, oocytes were aspirated from cow ovaries and allowed to mature before they were fertilized with bull semen. Embryos were cultured for five days and evaluated for signs of compaction (morula stage). Only embryos that showed evidence of compaction were further cultured for 72 hours before they were classified morphologically. On Day 8, embryos that showed a distinct inner cell mass and blastocoele were classified as 'blastocysts' while those that failed to transition from morula to blastocyst were classified as 'degeneratives'. Blastocysts (n = 20) and degeneratives (n = 20) were pooled and stored in RNAlater (Ambion, TX) to preserve RNA integrity. RNA was extracted from each embryo pool using RNaqueous Micro (Ambion), quality checked (Additional File 5) using Pico6000 on a Bioanalyzer 2100 (Agilent, CA), and amplified using MessageAmp II (Ambion). Sequencing libraries from amplified RNA (50 ng) for each pool was prepared according to Illumina's mRNA-seq protocol and sequenced on a Genome Analyzer IIx at the University of Wisconsin Biotechnology Center by one lane of a 75 bp single end run and one lane of a paired end 81 bp run. Because paired end reads were intrinsically associated, we defined a 'fragment' as a cDNA fragment that was sequenced from either end. Thus, there were two sequencing reads for one fragment in a paired end run and one sequencing read for one fragment in a single end run. Sequencing data in fastq format can be accessed by GEO with the accession number GSE25082.

### Alignment of sequencing reads to the bovine reference genome

The alignment of sequencing reads to the bovine reference genome (repeat masked assembly Btau4 downloaded from UCSC genome browser) comprised three stages. In the first stage, sequencing reads from blastocysts and degenerative embryos were trimmed to 60 bp and mapped independently using the software package Tophat v1.0.13 [11]. Tophat uses Bowtie [12] for alignment and is able to detect novel splice junctions by splitting reads to segments and joining segment alignments [11]. A total of 109,929 putative novel splice junctions were discovered from the data. Each junction was supported by at least one spliced read with at least five bases aligned to either exon. In the second stage of the alignment, putative novel junctions and 186,074 known junctions compiled from the Ensembl gene predictions and Ref-Seq gene models were combined and supplied to Tophat, which then aligned reads separately. This mapping strategy was able to cope with data sets with different read lengths and to fully utilize the identified novel junctions from all samples. A maximum of one mismatch in each of the 20 bp segments was allowed. Multireads that mapped equally well to more than 20 genomic locations were discarded. In the third stage, all unmapped reads were collected to be examined for evidence of polyadenylation at the ends. Because the sequencing was not stranded, we looked for reads that contained at least six As at the 3'end or six Ts at the 5'end. These polyA reads were trimmed and only reads with remaining sequences at least 25 bp long were analyzed further. We obtained 534,090 and 862,915 polyA reads in blastocysts and degeneratives, respectively (Table 1). These reads were mapped to the reference genome using the software package Bowtie allowing up to one mismatch and forcing uniqueness [12]. We further filtered out genomic alignments where at least six consecutive nucleotides downstream of the polyA cleavage sites were As. Cleavage sites within 15 bp of each other were considered to result from the same putative polyA sites. We considered the most frequent polyA signals (AAUAAA and AUUAAA) that occurred within 50 bp of the cleavage sites.

### Digital gene expression and expression microarrays

For digital gene expression measurements, we first obtained constitutive exons that are present in all isoforms of each of the genes, and then filtered out exons that overlapped between genes. These non-overlapping constitutive exon sets were then used to calculate overall gene expression with assistance from SAMtools [22] and BEDTools [23]. Raw counts were quantile normalized using a function in the 'limma' package in R [24]. For differential gene expression analysis with count data using a negative binomial distribution without replication, the DESeq package in R was used [14]. Microarray data was obtained in a previous study (GEO accession: GSE24936) and normalized as described in Huang et al. [9]. For Gene Ontology enrichment analysis, GO annotations were obtained from the 'org.Bt.eg.db' database from R/Bioconductor for uniquely identified genes. GO enrichment was tested using a hypergeometric test implemented in the 'GOstats' package in R [25].

### Assembly of transcriptome

The alignments were assembled using Scripture, which assembles transcripts at disjoined genomic locations by finding paths through a connectivity graph representation of the alignments [15]. After constructing all possible paths, Scripture then identifies statistically significant paths that deviated from the background distribution.

### Identification of novel transcriptional activity

To identify novel transcriptional activities that have not been annotated, we required that for a locus that was farther than 1000 bp away from known genes, at least one assembled transcript was at least 500 bp long and covered by at least 40 sequencing fragments. Our sequencing fragments had a mean insert size of 122 bp, this threshold for expression was about five fragments per base. Transcripts that were overlapped by repeats for more than 50% of the sequences were discarded. The Cuffcompare utility in the Cufflinks package was used for comparing transcripts to known annotations [21]. Regions that passed our filters were considered novel transcribed units (TUs). For external support of novel TUs, EST alignments and PhastCons scores were downloaded from the UCSC genome browser and compared to novel TUs using BEDTools [23]. We required a valid support by EST alignments to be at least 50% coverage.

### Analysis of alternative splicing events

We estimated and classified alternative splicing events from reads that mapped to exon-exon junctions [26]. We conservatively distrusted reads mapped to intronic regions and assembled first or last exons so we classified only the most frequent three classes of alternative splicing, "exon skipping", "alternative 5' splice site" and "alternative 3' site". For "exon skipping", we required that all three junctions be supported by at least two sequencing fragments. For "alternative 5' splice site" and "alternative 3' splice site" classes, we required that junctions be supported by at least two sequencing fragments in addition to 100% coverage of bases between the alternative splice sites. To test for association between alternative splicing and developmental status of embryos, Fisher's exact tests were used. For exon skipping, the count for inclusion of the exon was calculated as the average of the two splice junctions involving the exon.

## Authors' contributions

WH and HK designed the study and wrote the manuscript. WH performed experiments and analyzed the data. Both authors read and approved the final manuscript.

## Supplementary Material

Additional file 1 **List of differentially expressed genes and novel TUs identified by DESeq**. Click here for file

Additional file 2 **RNA-seq is more sensitive than microarrays**. Histogram of RNA-seq gene expression was plotted according to MAS5 Present/Marginal/Absent calls for blastocysts (a) and degeneratives (b). Click here for file

Additional file 3 **Test for DNA contamination in aRNA samples**. Click here for file

Additional file 4 **Intronic read fraction as a function of pre-mRNA fraction**. In this hypothetical example, exons were assumed to constitute 1/20 of the bases of genes. As pre-mRNA fraction increases, intronic read fraction also increases at a faster rate. Click here for file

Additional file 5 **Bioanalyzer quality check of RNA extracted from pools of embryos**. Approximately 5 ng of total RNA was analyzed on a RNA Pico6000 chip. Click here for file
