# Supplementary figures and images for: Comparison of transcriptomic landscapes of bovine embryos using RNA-Seq
Source: BMC Genomics. 2010 Dec 17;11:711. doi: 10.1186/1471-2164-11-711 (PMC3019235; doi:10.1186/1471-2164-11-711)

(a)

**Blastocyst**

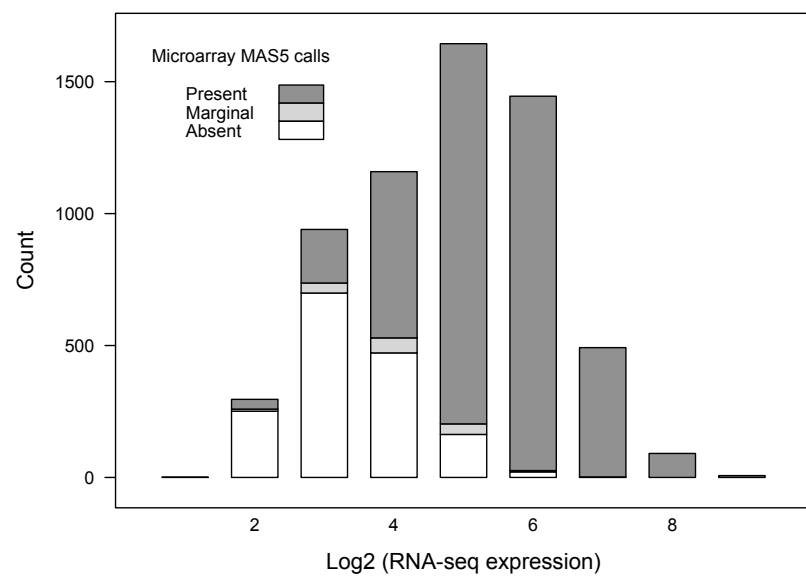

(b)

**Degenerative**

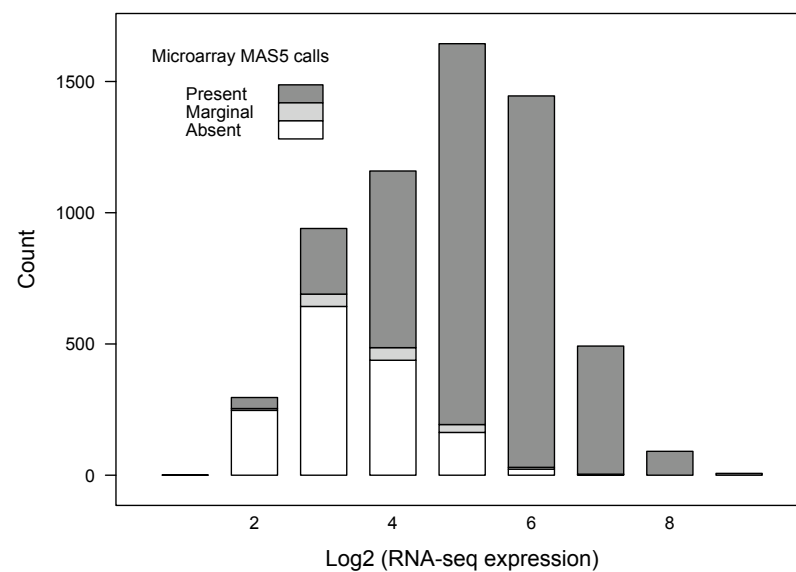

Supplement: Additional file 2 — RNA-seq is more sensitive than microarrays. Histogram of RNA-seq gene expression was plotted according to MAS5 Present/Marginal/Absent calls for blastocysts (a) and degeneratives (b). [file 1471-2164-11-711-S2.PDF]

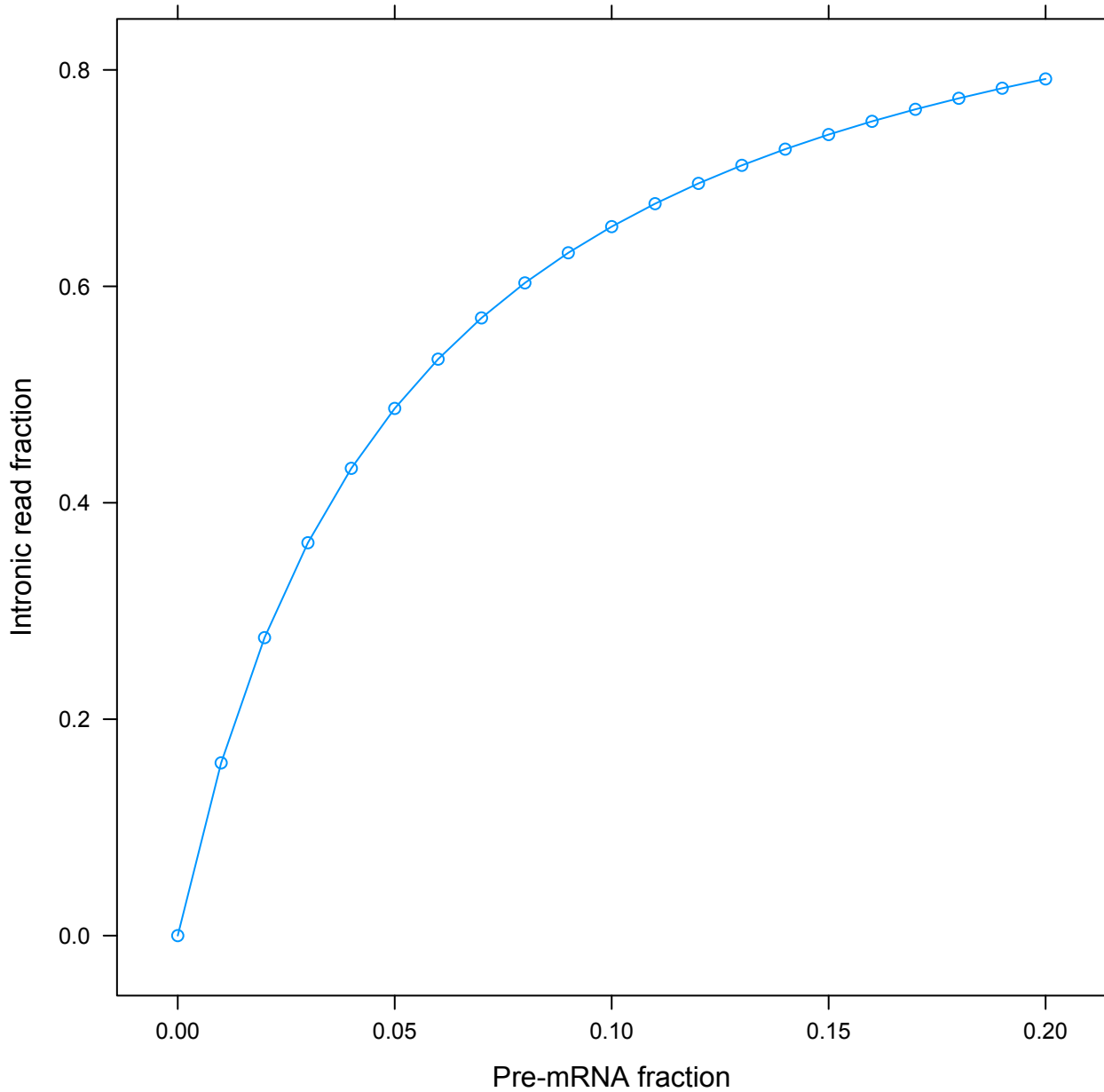

Supplement: Additional file 4 — Intronic read fraction as a function of pre-mRNA fraction. In this hypothetical example, exons were assumed to constitute 1/20 of the bases of genes. As pre-mRNA fraction increases, intronic read fraction also increases at a faster rate. [file 1471-2164-11-711-S4.PDF]

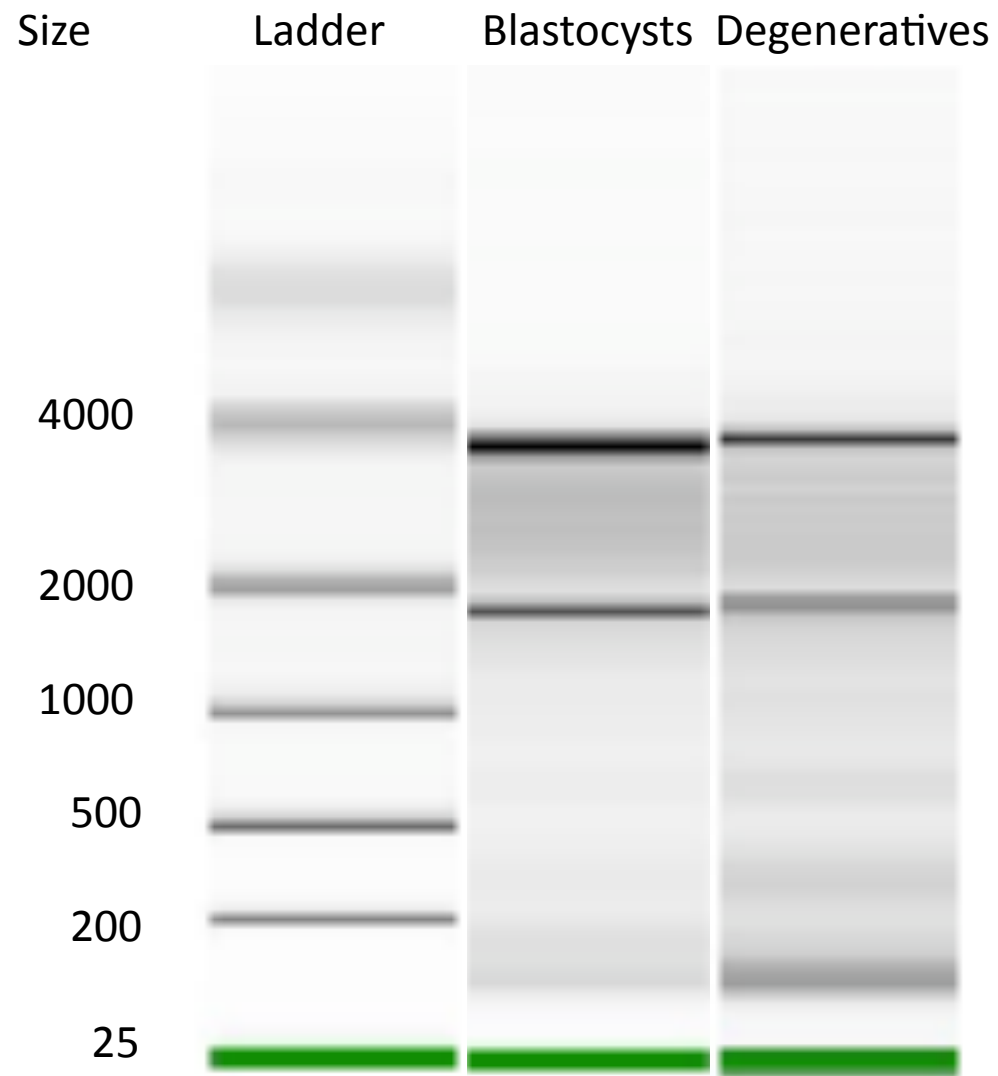

Supplement: Additional file 5 — Bioanalyzer quality check of RNA extracted from pools of embryos. Approximately 5 ng of total RNA was analyzed on a RNA Pico6000 chip. [file 1471-2164-11-711-S5.PDF]
