# Supplementary material for: Comparison of transcriptomic landscapes of bovine embryos using RNA-Seq
Source: BMC Genomics. 2010 Dec 17;11:711. doi: 10.1186/1471-2164-11-711 (PMC3019235; doi:10.1186/1471-2164-11-711)
Supplement: Additional file 3 — Test for DNA contamination in aRNA samples. [file 1471-2164-11-711-S3.PDF]

Test for presence of DNA in aRNA samples

To test for possible DNA contamination in the aRNA samples, we randomly selected three regions located in three different chromosomes where no known gene was found and one region located in an expressed gene PHLDA2. The primers in PHLDA2 were designed to contain both exonic and intronic regions, making them specific for DNA or pre-mRNA. PCRs were performed to detect presence of DNA in the aRNA and cDNA samples using primers in the following table. The amount of cDNA and aRNA used for PCR was equivalent to 1ng starting aRNA. For positive controls, we also included three concentrations of DNA (1ng, 0.1ng, 0.01ng). PCR was performed using the GoTaq Flexi DNA polymeriase (Promega, Madison, WI) with manufacture supplied buffer and 1.5mM $MgCl_2$, 0.2mM each dNTP, 0.5 μM each primer and 1.5U Taq polymerase in a 25 μL reaction. The cycling condition was 94 °C for 5 min, and 38 cycles of 94 °C for 30 s, 55 °C for 30 s, and 72 °C for 45 s, followed by a final extension of 72 °C for 7 min.

The results clearly indicated the absence of DNA at detectable level (<= 0.01ng or <= 0.1ng, depending on genomic regions) in the aRNA samples (See figure below).

Table: Genomic locations and primer sequences

Type	Genomic region	Forward primer	Reverse primer	Size (bp)
Intergenic	chr1:50017934+50018924	CTACTGTGGCCATGGAAGGT	TGGGAACTGGGTAAAACAGC	991
Intergenic	chr10:49162605+49163532	TCAGCACAAGGTCACTGAGG	ACCACCAGCTGATGCTTTCT	928
Intergenic	chr20:49890236+49891174	TAAGATGGCCCCTGATCAAA	ATTCCCCTTGTGCAGGTATG	939
PHLDA2	chr29:50555625+5055584	CACAGCTCCCTGAATCCTTC (intronic)	GTAGCTAATTATGTAGCTTG (exonic)	219

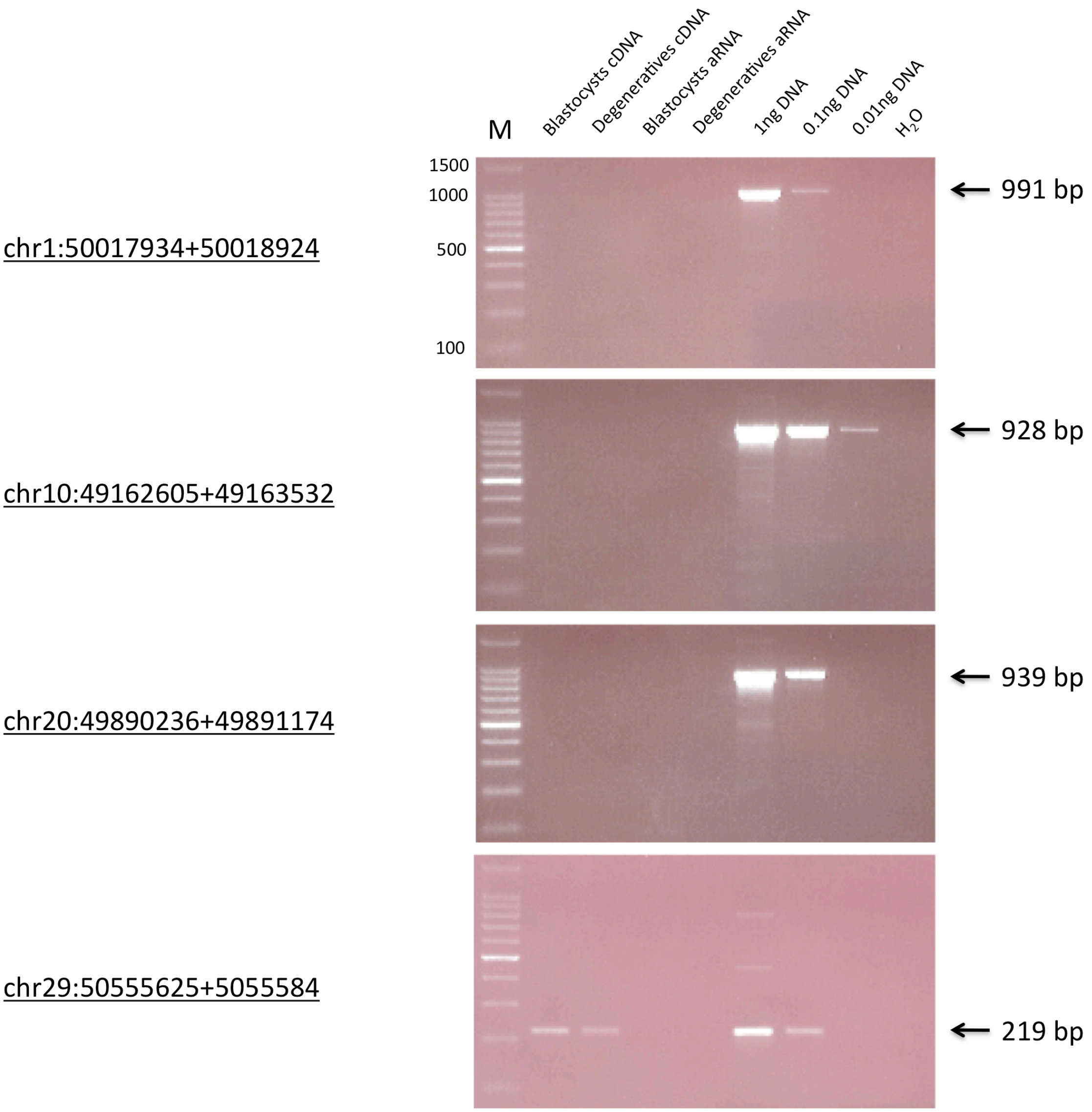

Figure: Gel picture of PCR for testing presence of DNA in aRNA samples.
